# Rheological and Microstructural Characterization of Steel Slag Powder-Modified Asphalt Mastics: Insights into High-Temperature Performance Enhancement

**DOI:** 10.3390/ma18061357

**Published:** 2025-03-19

**Authors:** Xiaodong Xie, Jie Gao, Zongjie Yu, Liang Song, Xuzhi Zhu

**Affiliations:** 1 Xinjiang Academy of Transportation Sciences Co., Ltd., Urumqi 830000, China; xiaodong3056@163.com (X.X.); 13511844380@163.com (Z.Y.); 15001656750@163.com (X.Z.); 2 Key Laboratory of China’s Transportation Industry for Highway Engineering Technology in Arid Desert Region, Urumqi 830099, China; 3 School of Civil Engineering and Architecture, East China Jiaotong University, Nanchang 330013, China; 4 Xinjiang Transpottation Planning Survrying and Design Institute Co., Ltd., Urumqi 830006, China; slpl@sohu.com; 5 School of Traffic and Transportation Engineering, Xinjiang University, Urumqi 830049, China

**Keywords:** steel slag powder, asphalt mastic, rheological performance, MSCR, 2D-COS

## Abstract

This study systematically investigates the rheological modification mechanism of steel slag powder (SSP) as an alternative filler in asphalt mastics, with comparative analysis against conventional limestone powder (LP). Four filler-to-asphalt (F/A) ratios (0.6–1.2) were employed to prepare modified mastics. Comprehensive characterization through laser diffraction analysis, BET nitrogen adsorption, and scanning electron microscopy (SEM) revealed SSP’s significant microstructural advantages: a 29.2% smaller median particle size (D50) and 7.06% larger specific surface area compared to LP, accompanied by enhanced interparticle connectivity and morphological complexity. Rheological evaluation via dynamic shear rheology (DSR) demonstrated SSP’s superior performance enhancement—particularly at elevated F/A ratios (1.0–1.2), where multiple stress creep recovery (MSCR) tests showed a 6.9–46.06% improvement in non-recoverable creep compliance (*J_nr_*) over LP-modified counterparts. The temperature sweep analysis indicated SSP’s effectiveness in reducing the temperature susceptibility index by 9.37–18.06% relative to LP. Fourier-transform infrared spectroscopy (FTIR) combined with two-dimensional correlation analysis (2D-COS) confirmed the dominance of physical interactions over chemical bonding in the SSP–asphalt interface. The results establish SSP’s dual functionality as both a rheological modifier and sustainable construction material, providing mechanistic insights for optimizing steel slag utilization in pavement engineering.

## 1. Introduction

With the rapid development of road infrastructure, extending the service life of asphalt pavements has emerged as a critical research focus in highway engineering. In high-temperature regions, asphalt pavements are prone to forms of distress such as rutting, shoving, and bleeding [1,2,3], prompting researchers to incorporate modifiers (e.g., crumb rubber (CR) and styrene–butadiene–styrene (SBS)) for enhancing the aging resistance and rutting performance of asphalt binders at elevated temperatures [4,5]. However, conventional polymer modifiers face challenges including high costs and reliance on non-renewable resources, thereby necessitating innovative strategies to reduce the modifier consumption while improving high-temperature performance [6,7]. Concurrently, the steel industry generates substantial steel slag waste due to its accelerated growth, with improper stockpiling of this by-product causing severe environmental contamination [8]. Therefore, achieving efficient resource utilization of steel slag has become an urgent scientific and engineering priority [9,10,11].

Current research indicates that the utilization of steel slag in China remains suboptimal compared to developed regions (e.g., Europe, the U.S., and Japan), with substantial unprocessed stockpiles exacerbating environmental contamination [12]. While steel slag has been predominantly utilized as an aggregate in hydraulic concrete to enhance the mechanical integrity of structural layers [13,14], its application in asphalt mixtures and pavement bases demonstrates superior physicochemical compatibility, effectively improving the high-temperature rutting resistance and moisture-damage resistance as a sustainable alternative to conventional aggregates [15,16]. Nevertheless, the long-term stability of slag-incorporated pavements is compromised by intrinsic limitations, including complex chemical compositions, potential heavy metal leaching [17], and volumetric expansion-induced cracking that shortens the service life. This dichotomy underscores the urgent need to optimize steel slag deployment in road engineering. Concurrently, the overexploitation of LP—the predominant filler in asphalt mixtures—has triggered irreversible ecological degradation [18]. In this context, SSP, an industrial by-product characterized by high yield and processability, emerges as a dual-benefit solution: its substitution for LP not only mitigates mineral resource depletion but also establishes an eco-efficient pathway for steel slag valorization [19,20,21]. Compared with limestone powder (LP), steel slag powder (SSP) exhibits a larger specific surface area and more intricate internal void structures, enabling it to adsorb a greater amount of asphalt components and thereby enhance the performance of asphalt mortar [13,22,23]. Furthermore, the finer particle morphology of SSP relative to LP amplifies the filling effect within asphalt, contributing to improved stability of the asphalt mortar system [8]. To elucidate the modification mechanism of SSP in asphalt, researchers employed scanning electron microscopy (SEM) and revealed that the complex interlocking particle morphology of SSP facilitates enhanced asphalt adhesion, which strengthens the interfacial bonding between asphalt and aggregates [24]. X-ray diffraction (XRD) analysis identified mineral constituents in SSP, such as quartz and bauxite. These minerals engage in physiochemical interactions with organic components in asphalt, leading to optimized material properties [25]. Additionally, Fourier-transform infrared (FTIR) spectroscopy demonstrated that the incorporation of SSP effectively suppresses asphalt oxidation, thereby enhancing its aging resistance [20,26].

In summary, the incorporation of SSP into asphalt mixtures demonstrates potential for enhancing both high-temperature rutting resistance and moisture stability. Building upon the observed effects of SSP on asphalt binders and mastics, this study systematically investigates the rheological behavior of SSP-modified asphalt mastics under elevated temperatures, where SSP replaces conventional LP as the filler. This substitution strategy not only reduces the exploitation of natural limestone resources but also alleviates environmental pollution caused by steel slag stockpiling. To elucidate the underlying mechanisms, laser granulometry, Brunauer–Emmett–Teller (BET) adsorption analysis, and scanning electron microscopy (SEM) were employed to characterize the microstructural disparities between SSP and LP. Asphalt mastics with four filler-to-binder (F/A) ratios (0.6–1.2) were formulated using SSP-blended asphalt, while LP-based mastics served as the control group. The high-temperature rheological performance was rigorously evaluated through dynamic shear rheometer (DSR) tests and multi-stress creep recovery (MSCR) assays. Furthermore, two-dimensional correlation spectroscopy (2D-COS) was applied to decode functional group evolution and adsorption mechanisms derived from Fourier-transform infrared (FTIR) spectra. Future research should focus on optimizing the SSP dosage, surface modification protocols, and asphalt–SSP interfacial interactions, which are pivotal for advancing high-performance pavement materials and fostering sustainable development in road engineering.

## 2. Materials and Methods

### 2.1. Materials

#### 2.1.1. Asphalt

This study utilizes 90# matrix asphalt provided by the Karamay Refinery in Xinjiang. The technical specifications and laboratory performance test results are presented in Table 1, according to the Standard Test Methods of Bitumen and Bituminous Mixtures for Highway Engineering (JTG E20-2011) [27].

#### 2.1.2. Fillers

Traditional LP and SSP were selected as fillers. The LP was provided by a company based in Urumqi, Xinjiang, while the SSP was prepared by the Key Laboratory of Highway Engineering and Transportation in Arid and Desert Areas. The basic performance characteristics of both fillers are summarized in Table 2, according to the Test Methods of Aggregates for Highway Engineering (JTG 3432—2024) [28].

#### 2.1.3. Asphalt Mastic

**Figure 1:**
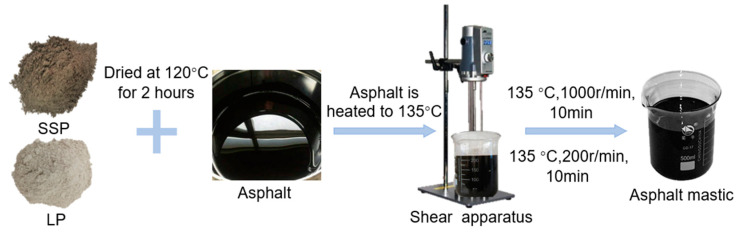
Flow chart of specimen preparation.

This study investigates the preparation of asphalt mastics using SSP, LP, and 90# matrix asphalt at four distinct F/A ratios. The relevant test conditions are detailed in this standard [27]. The preparation process is depicted in Figure 1 and outlined below:

1. The measured 90# matrix asphalt was heated in an oven at 135 °C for 2 h to achieve a fluid state. Meanwhile, predetermined proportions of LP and finely ground SSP were heated in an oven at 120 °C for 2 h. Both the asphalt and fillers were maintained at a constant temperature for an additional 30 min;
2. The heated asphalt was then transferred to the oil bath of a high-speed shear mixer, with temperature control maintained throughout the process. Subsequently, the preheated LP and SSP were added to the asphalt sequentially, while continuously stirring with a glass rod to ensure a uniform dispersion of the fillers. The mixture was subjected to high-speed shearing at 135 °C (1000 rpm) for 10 min, followed by low-speed shearing (200 rpm) for an additional 10 min to eliminate entrained air bubbles;
3. After preparation, the mixture was placed in an oven at 135 °C for 30 min to eliminate any remaining air bubbles and ensure complete curing.

In the following text, the LP-modified asphalt mastic and SSP-modified asphalt mastic are represented as LMA and SMA, respectively.

### 2.2. Methods

#### 2.2.1. PSD

PSD is widely employed to assess nanoparticles. Mineral powder particles generally display a size distribution within a defined range, commonly referred to as the particle size distribution. The primary parameters used to characterize this distribution are D10, D50, D90, and specific surface area. In this study, the differences in PSD and SSA between SSP and LP were measured using a Dandong Baite BT-9300LD laser particle size analyzer produced by Dandong Baite Instrument Co., Ltd., Dandong, Liaoning Province, China.

#### 2.2.2. BET

The BET test determines the specific surface area, pore volume, and pore size distribution of solid materials based on the behavior of physical adsorption–desorption and the quantity of adsorbed gas. In this study, nitrogen (N_2_) adsorption experiments were conducted using a Micromeritics ASAP 2460 fully automated surface area and porosity analyzer (manufactured by Micromeritics, Norcross, GA, USA) to evaluate the adsorption isotherms and internal pore size distributions of the two fillers (LP and SSP).

#### 2.2.3. SEM

Microstructural analysis was performed using a ZEISS Gemini SEM 300 scanning electron microscope (Carl Zeiss AG, Oberkochen, Germany). This study investigated the micro-morphological characteristics of LP and SSP to determine the influence of these fillers on asphalt adhesion. Magnifications of 10,000× and 40,000× were used.

#### 2.2.4. DSR

The high-temperature performance of LMA and SMA was measured using a DHR-20 dynamic shear rheometer produced by TA Instruments, New Castle, DE, USA. During the temperature sweep, the temperatures were set at 46, 52, 58, 64, 70, and 76 °C, with a fixed loading frequency of 10 rad/s. A parallel plate geometry with a diameter of 25 mm and a gap of 1 mm was utilized.

#### 2.2.5. MSCR

The MSCR test was employed to simulate the stress–strain behavior of the two asphalt binders under realistic pavement conditions. In this study, the test temperature was maintained at 60 °C, with applied stress levels of 0.1 kPa and 3.2 kPa in two distinct phases. Each phase included 10 cycles of repeated loading and recovery.

#### 2.2.6. FTIR

A Thermo Fisher Scientific Nicolet IS 20 FTIR Fourier infrared spectrometer (Thermo Fisher Scientific, Waltham, MA, USA) was selected to obtain the infrared spectra of different asphalt mortar specimens within a specific wavelength range. This spectrometer was also used to compare and analyze the characteristic functionalities to investigate whether any chemical reactions occurred during the modification process of asphalt mortar. The test wavelength range was 4000–400 cm^−1^ with a resolution of 4 cm^−1^.

## 3. Results and Discussion

### 3.1. Physical Characteristics of Fillers

#### 3.1.1. Particle Size Distribution Characterization

**Figure 2:**
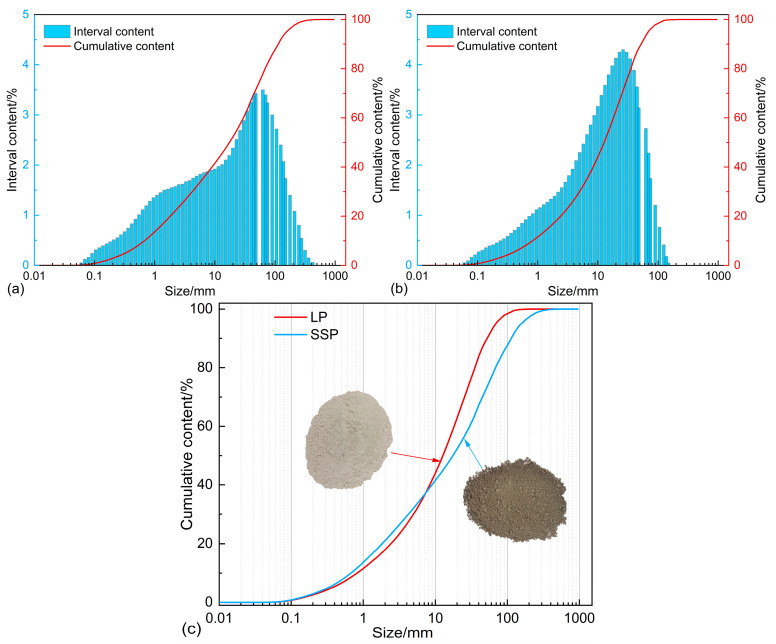
Particle size distribution: (**a**) LP; (**b**) SSP; (**c**) comparison of particle size distribution.

As shown in Table 3 and Figure 2, both SSP and LP exhibit a single peak in their particle size distribution. This indicates that the processing of these two powders is well-controlled, resulting in uniform particle sizes that meet the practical requirements for engineering applications. The filler plays a crucial role in the interaction between the asphalt and aggregates, which directly influences the performance of the asphalt mastic. Studies have shown that over 60% of asphalt adsorption behavior in asphalt mixtures occurs on filler particle surfaces, highlighting the critical role of specific surface area in asphalt adsorption. Furthermore, Guo et al. [29] demonstrated that the specific surface area of filler particles plays a dominant role in asphalt adsorption behavior compared to their surface mineral properties.

#### 3.1.2. Micro-Morphological Characteristics

**Figure 3:**
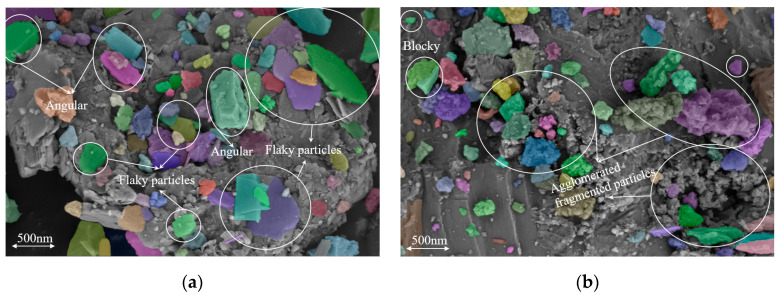
SEM images: (**a**) LP; (**b**) SSP.

As shown in Figure 3, there are significant differences in the microstructures of LP and SSP. From a morphological perspective, LP particles are relatively large and are predominantly covered by angular or flaky particles. The particle distribution is fairly uniform and the surface is smooth, with few spherical agglomerates. However, there is some degree of overlap and aggregation between the particles. In contrast, SSP particles exhibit irregular shapes, including blocky, agglomerated, and fragmented particles, with a non-uniform particle size distribution. The surface of the SSP particles is rough, featuring cracks and uneven topography.

From the analysis of the microporous structure, the voids between LP particles are relatively small and uniformly distributed, indicating good filling performance. In contrast, the voids between SSP particles are larger. The agglomeration of particles leads to the formation of significant micropores. This structural characteristic may result in a higher number of capillary pores in the matrix, thereby increasing the porosity.

#### 3.1.3. Characterization of Internal Pore Structure

**Figure 4:**
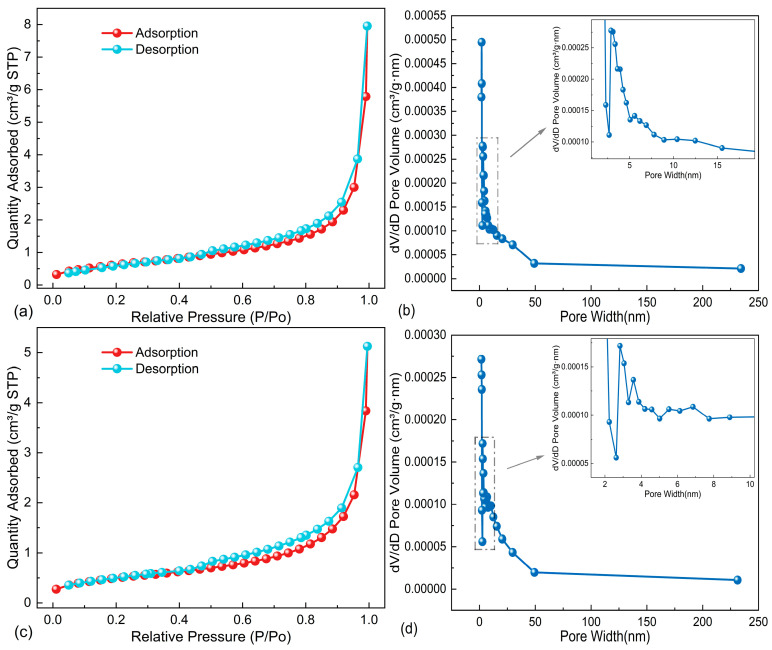
Adsorption–desorption curves of two fillers: (**a**) adsorption curve of SSP; (**b**) pore distribution of SSP; (**c**) adsorption curve of LP; (**d**) pore distribution of LP.

Nitrogen adsorption experiments were conducted to analyze the micropore distribution and micro-void structure of the SSP and LP. The results are presented in Figure 4 and Table 4. As shown in Figure 4a,c, both SSP and LP exhibit low nitrogen adsorption, with a slow increase in adsorption within the low-pressure region. This indicates a limited number of fine voids in both powders. Notably, the adsorption and desorption curves for LP show a significant gap in the medium-to-high pressure range. This suggests that the pores between LP particles are predominantly narrow, elongated, and exhibit a layered stacking structure. In contrast, the gap between the adsorption and desorption curves of SSP is smaller within the same pressure range, indicating that the pores in SSP are primarily formed by particle-to-particle stacking. This finding is consistent with the SEM morphological results. Additionally, at equivalent pressures, the nitrogen adsorption capacity of SSP exceeds that of LP.

The specific surface area, mesopore-specific surface area, average pore volume, average pore diameter, and pore size distribution curves of the two fillers were calculated from the adsorption–desorption isotherms. As shown in Table 4 and Figure 4b,d, the overall pore volume of both SSP and LP fluctuates and decreases as the particle size increases. The pore size distribution trends for both fillers are generally similar, each exhibiting a single peak; however, the peak of the pore size distribution curve for SSP is higher than that for LP.

### 3.2. Results of DSR

#### 3.2.1. Complex Modulus

**Figure 5:**
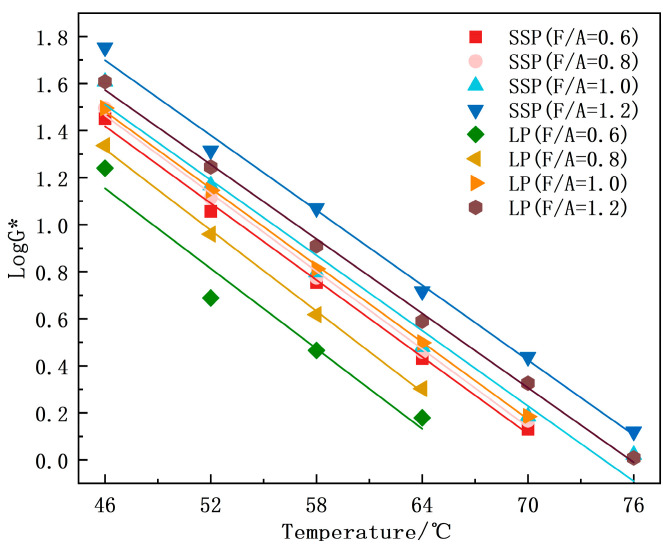
G* of asphalt mastics.

The temperature sweep results for the asphalt mastics are presented in Figure 5. Both types exhibited a decrease in complex modulus (G*) as the temperature increased, which is consistent with the transition from a linear elastic to a viscous flow state during heating. At identical temperatures, SMA (F/A = 1.2) demonstrated the highest Log(G*) values. Notably, SMA consistently exhibited higher Log(G*) values than LMA at equivalent F/A ratios, indicating that SSP modification enhances the high-temperature performance of asphalt mastics by improving deformation resistance compared to LP-modified counterparts.

At 46 °C, the Log(G*) of SMA increased by 17.03%, 11.90%, 7.55%, and 9.02% compared to LMA under the four different F/A ratios. At 52 °C, the increases were 53.53%, 16.04%, 1.85%, and 5.66%, respectively. At 58 °C, the corresponding improvements were 62.09%, 25.43%, 1.92%, and 17.56%. At 64 °C, the increases were 71.05%, 51.51%, 3.81%, and 21.05%, respectively. These results demonstrate that SMA exhibits superior resistance to high-temperature deformation compared to LMA. Therefore, replacing LP with SSP as a filler enhances the high-temperature deformation resistance of asphalt mastic, which aligns with the findings reported by Guo et al. [30].

This observation highlights that SMA consistently shows superior high-temperature deformation resistance. The enhanced performance can be attributed to SSP’s larger specific surface area and more intricate porous microstructure, which facilitate the preferential adsorption of polar components in asphalt [29]. This selective adsorption reduces the light fractions while increasing the heavy fraction proportions, thereby inducing asphalt hardening and improving the mastic’s deformation resistance. In addition to the adsorption effects attributed to its specific surface area, SSP further enhances the structural stability of asphalt mastic through its superior stiffness, thereby improving the high-temperature deformation resistance of the material [17].

Notably, both asphalt mastics showed progressively enhanced high-temperature deformation resistance with increasing F/A ratios across the four investigated levels. At the same F/A ratio, the high-temperature effective temperatures of SMA were consistently higher than those of LMA. The high-temperature effective temperatures of the two asphalt binders under different F/A ratios, ranked from highest to lowest, were as follows: SMA (F/A = 1.2) > LMA (F/A = 1.2) > SMA (F/A = 1.0) > LMA (F/A = 1.0) > SMA (F/A = 0.8) > SMA (F/A = 0.6) > LMA (F/A = 0.8) > LMA (F/A = 0.6).

#### 3.2.2. Rutting Factor

**Figure 6:**
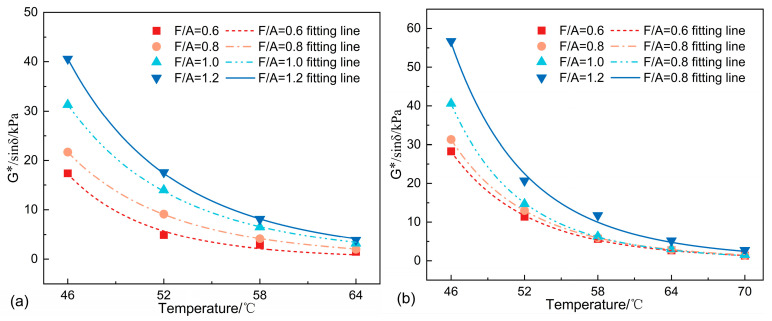
G*/sin*δ* of different asphalt mastics: (**a**) LP, G*/sin*δ*; (**b**) SSP, G*/sin*δ*.

**Figure 7:**
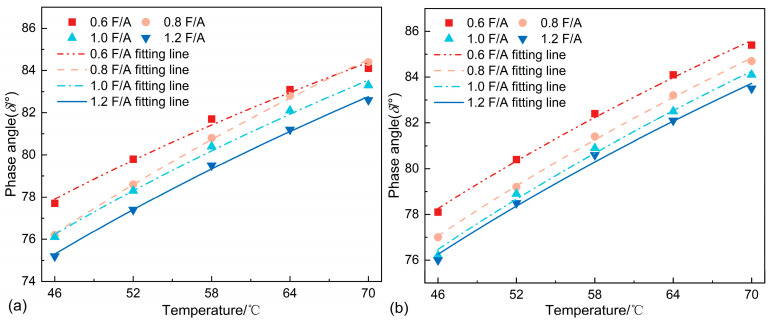
The *δ* of different asphalt mastics: (**a**) LP, *δ*; (**b**) SSP, *δ*.

The G*/sin*δ* is a widely adopted parameter in DSR tests for evaluating the high-temperature rheological properties of asphalt binders, specified in standards such as AASHTO M 332 [31] and ASTM D7175 [32]. The calculated G*/sin*δ* values of asphalt mastics are presented in Figure 6. The results reveal a positive correlation between the G*/sin*δ* and F/A ratios, indicating enhanced rutting resistance with increased filler content. At equivalent F/A ratios, SMA consistently demonstrated higher G*/sin*δ* values than LMA, suggesting SSP’s superior effectiveness in improving rutting resistance. This enhancement mechanism aligns with SSP’s deformation resistance improvement, where its incorporation reduces mastic fluidity. Notably, the G*/sin*δ* escalation followed a non-linear pattern with increasing filler content, exhibiting rapid escalation. Figure 7 displays the temperature-dependent *δ* results. The observed *δ* reduction with F/A increments demonstrates modified viscoelastic properties induced by the SSP and LP incorporation. Comparative analysis under identical F/A and temperature conditions reveals that SSP exerts a more pronounced influence on viscoelastic behavior than LP.

#### 3.2.3. Temperature Sensitivity Analysis

The temperature sensitivity of the asphalt mastics was evaluated using the storage modulus (G′) and loss modulus (G″). Temperature sensitivity critically governs the performance of asphalt mixtures, where heightened sensitivity adversely impacts low-temperature cracking resistance and permanent deformation characteristics. Univariate linear regression analyses were performed on the logarithmic-scale temperature dependence of G′ and G″. The absolute slopes of these regression lines, which are proportional to the temperature sensitivity of the asphalt mastic, served as quantitative indicators of thermal susceptibility.

**Figure 8:**
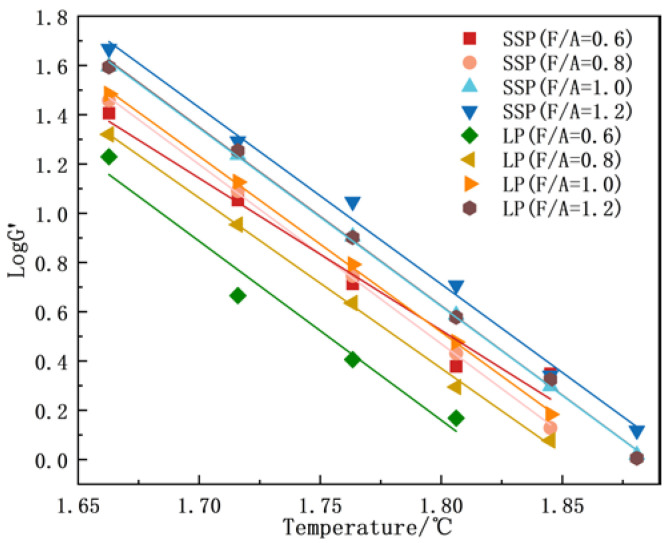
G′ of different asphalt mastics.

**Figure 9:**
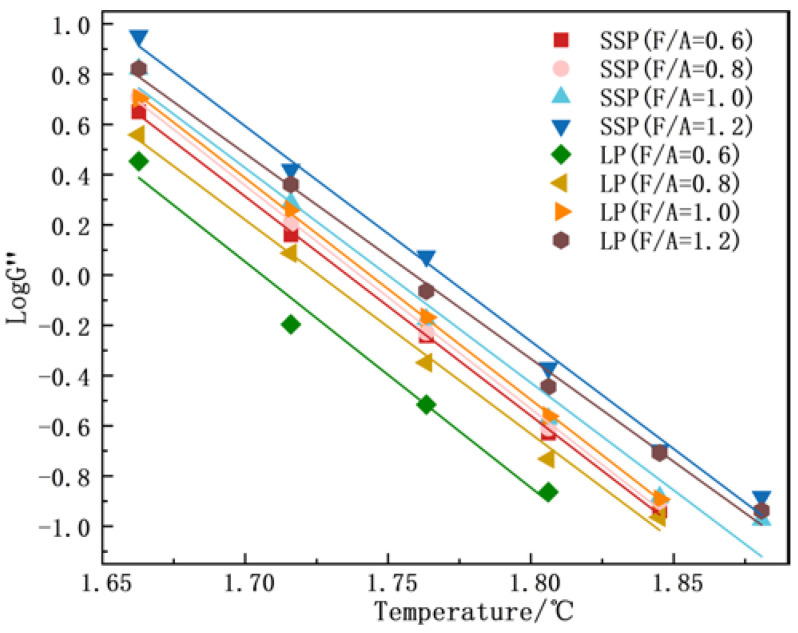
G″ of different asphalt mastics.

As evidenced by the linear regression analyses in Figure 8 and Figure 9 and Table 5 and Table 6, increasing the F/A ratio reduced the temperature sensitivity of asphalt mastics, which is consistent with the findings of Meng et al. [8]. Furthermore, the comparative analyses between SMA and LMA at identical F/A ratios revealed that SSP incorporation significantly attenuated temperature sensitivity, with reductions of 9.37%, 13.38%, 14.01%, and 18.06% observed across the four distinct F/A ratios. These findings demonstrate that the strategic substitution of LP with SSP effectively mitigates temperature sensitivity in asphalt mastics, thereby enhancing their applicability in regions subjected to extreme summer temperature fluctuations.

#### 3.2.4. Results of MSCR

**Figure 10:**
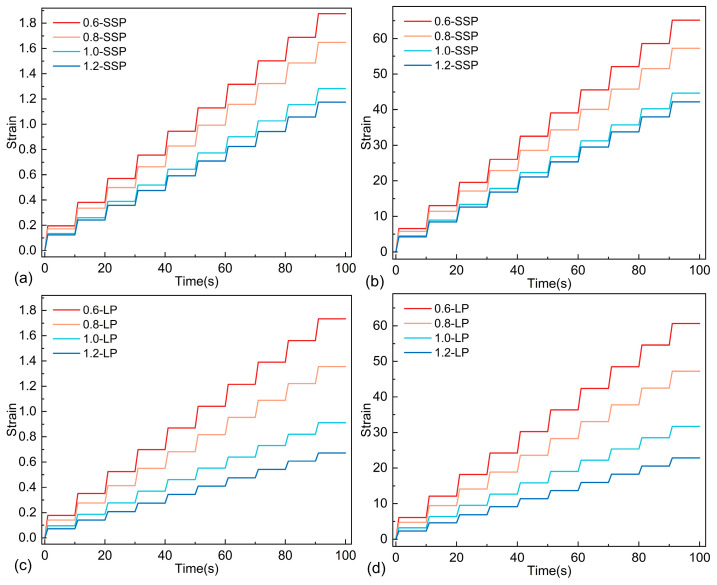
MSCR test results: (**a**) SMA, 0.1 kPa; (**b**) SMA, 3.2 kPa; (**c**) LMA 0.1 kPa; (**d**) LMA 3.2 kPa.

**Figure 11:**
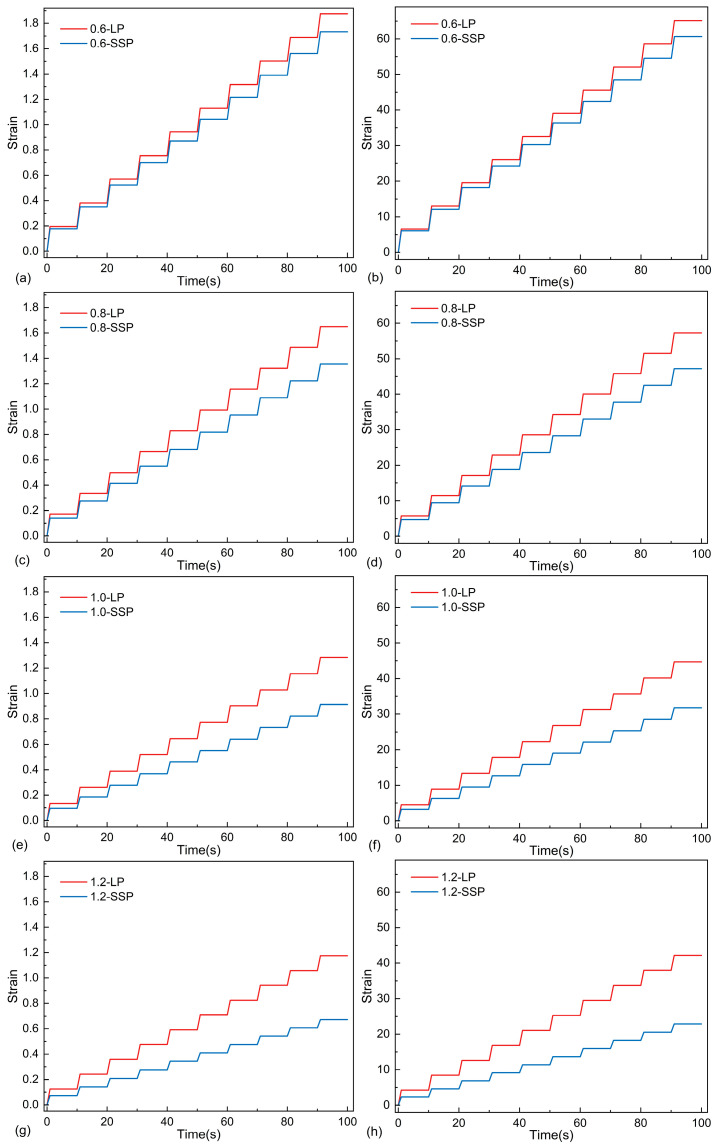
MSCR test results under different F/A ratios: (**a**) F/A = 0.6, 0.1 kPa; (**b**) F/A = 0.6, 3.2 kPa; (**c**) F/A = 0.8 0.1 kPa; (**d**) F/A = 0.6, 3.2 kPa; (**e**) F/A = 1.0, 0.1 kPa; (**f**) F/A = 1.0, 3.2 kPa; (**g**) F/A = 1.2, 0.1 kPa; (**h**) F/A = 1.2, 3.2 kPa.

The elastic recovery properties of the asphalt mastics were evaluated through non-recoverable creep compliance (*J_nr_*) and creep recovery rate (*R*) calculations, as illustrated in Figure 10 and Figure 11 and Table 7. The strain evolution patterns of both mastic systems are presented in Figure 10. A systematic reduction in strain values was observed with increasing filler content, demonstrating that filler incorporation significantly enhances the flow resistance of the asphalt mastics. This confirms that, within the tested dosage range, elevated mineral filler content improves permanent deformation resistance.

Comparative analysis of strain–time relationships for SSP- and LP-modified mastics under 0.1 kPa and 3.2 kPa stress levels revealed that LMA consistently exhibited lower cumulative strain than SMA. Notably, the strain differential between LMA and SMA became significantly greater with higher F/A ratios. These findings substantiate SSP’s capacity to enhance the high-temperature performance sustainability of asphalt mastics under prolonged thermal loading conditions.

The MSCR test evaluation metrics are presented in Table 7. Both modified asphalt mastics exhibited decreasing *J_nr_* with increasing F/A ratios, indicating enhanced high-temperature performance at higher filler contents. At equivalent F/A ratios, SSP demonstrated superior enhancement effectiveness over LP. Under 0.1 kPa stress loading, *J_nr_* reductions reached 8.47%, 17.68%, 28.21%, and 42.91% for SSP-modified mastics compared to LP-modified counterparts. Similarly, at 3.2 kPa stress loading, *J_nr_* decreased by 6.9%, 17.88%, 28.78%, and 46.06%, respectively.

Under 3.2 kPa stress loading, the measured *R* value exhibited a significant reduction compared to that under 1.0 kPa stress, which aligns with the observed engineering phenomenon that heavy-duty traffic conditions tend to induce more severe rutting deformation. No distinct correlation was established between *R_nr diff_* and filler content for both asphalt mastics, consistent with the irregular pattern observed in R values. Conversely, *J_nr diff_* demonstrated contrasting behaviors: the LMA showed a gradual decrease in *J_nr diff_* with increasing filler content, indicating enhanced resistance to stress sensitivity through mineral filler addition. In contrast, the SMA exhibited opposite characteristics, displaying progressively elevated stress sensitivity with higher SSP content.

Concurrently, the SMA specimen (F/A = 1.2) demonstrated optimal performance in both stress conditions, with non-recoverable creep compliance values of *J_nr_* _0.1_ = 0.668 kPa and *J_nr_* _3.2_ = 0.712 kPa, accompanied by peak creep recovery rates of *R*_0.1_ = 5.78 and *R*_3.2_ = 1.66. These results indicate relatively diminished elastic recovery capacity yet enhanced high-temperature permanent deformation resistance. This phenomenon further corroborates that increased SSP content reduces polar components within the asphalt matrix, thereby attenuating the elastic restoration capability of the asphalt mastic.

The experimental results indicate that the Jnr values for both asphalt mastics decrease significantly with an increase in the F/A ratio. It is evident that the addition of LP and SSP to the asphalt mastic results in a marked enhancement in overall strength compared to pure asphalt. To more accurately assess the change in overall strength induced by the addition of fillers, this study employs the strengthening effect index, *ΔJ_nr_*. This index quantifies the strength improvement in the asphalt mastic relative to pure asphalt, thereby highlighting the reinforcing effect of mineral fillers. The calculation formula is provided in Equation (1).

(1) $$∆J_{nr}=J_{nr}^{asphalt}-J_{nr}^{asphalt\;mastic}$$

where $J_{nr}^{asphalt}$ is the *J_nr_* value of the asphalt; $J_{nr}^{asphalt\;mastic}$ is the *J_nr_* value of the asphalt mastic.

**Figure 12:**
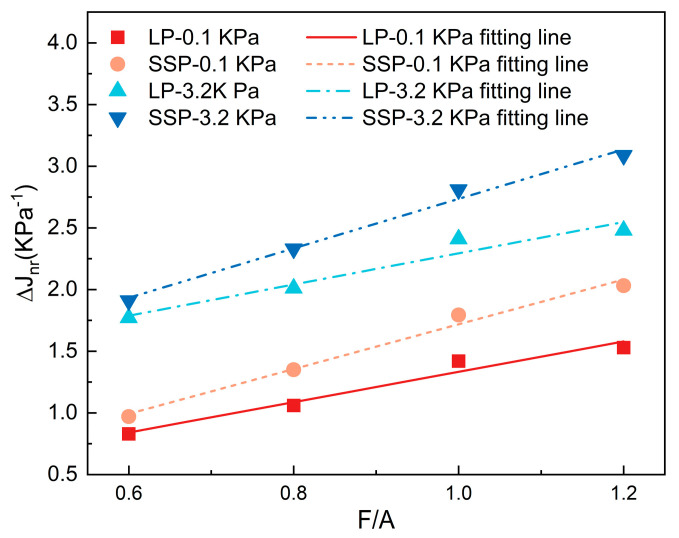
The Δ*J_nr_* results of different asphalt mastics.

The difference in *J_nr_* values between asphalt and its corresponding asphalt mastic, denoted as Δ*J_nr_*, serves as a quantitative indicator of the reinforcing effect of mineral fillers in the asphalt mastic. It also reflects the extent of strength improvement and enhancement in the high-temperature rheological properties in the asphalt mastic. A larger Δ*J_nr_* value indicates a more significant reinforcing effect of the mineral fillers, leading to improved high-temperature rheological performance. The results of Δ*J_nr_* for asphalt mastic samples, with varying mineral filler contents and different types of asphalt, are presented in Figure 12.

The reinforcing effect of mineral fillers in both asphalt mastics increases linearly with the F/A ratio under both 0.1 kPa and 3.2 kPa stress conditions. Under the 0.1 kPa stress condition, the reinforcing effect of SMA is 16.86%, 27.36%, 26.34%, and 32.81% higher than that of LMA at the four different filler contents, respectively. Similarly, under the 3.2 kPa stress condition, the improvements are 7.91%, 15.92%, 16.59%, and 24.52%, respectively. These findings indicate that SSP is more effective in enhancing the reinforcing effect of asphalt mastic than LP.

### 3.3. Results of FTIR

**Figure 13:**
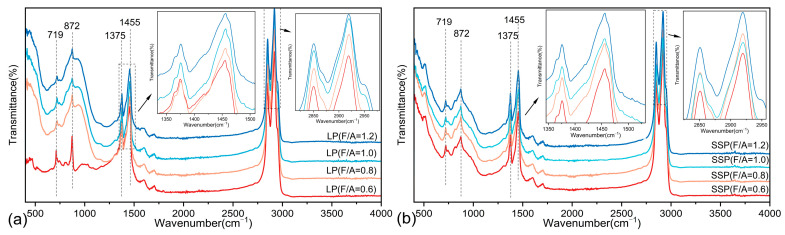
Evaluation of FTIR spectra of asphalt mortars at different F/A ratios: (**a**) LMA; (**b**) SMA.

The FTIR spectra of the two asphalt mastics are shown in Figure 13. Both the base asphalt and the two asphalt mastics show two strong absorption peaks at 2920 cm^−1^ and 2850 cm^−1^, corresponding to the asymmetric and symmetric stretching vibrations of -CH_2_- groups in orderly arranged long-chain alkyls [33]. The area of these peaks remains largely unchanged, suggesting that neither LP nor SSP influences the unsaturated hydrocarbons in the asphalt. Additionally, strong absorption peaks at 1456 cm^−1^ and 1375 cm^−1^—resulting from the scissoring vibration of methylene (-CH_2_-) and the symmetric bending vibration of methyl (-CH_3_-) groups—represent the relative content of free aliphatic compounds. Around 719 cm^−1^, an in-plane rocking vibration absorption peak of -CH_2_- groups is observed [34], reflecting the relative content of free long-chain aliphatic compounds. Comparing the ratios of peak areas at 1455, 1375, and 719 cm^−1^ to the total peak area in the 400–4000 cm^−1^ range for the two asphalt mastics reveals a gradual weakening of these peaks with increasing filler content. This trend indicates a reduction in the relative content of aliphatic compounds within the asphalt mastic as the filler content increases, which results in a higher proportion of heavy components, thereby explaining the enhanced high-temperature performance of asphalt mastic with LP and SSP.

As shown in Figure 13, the peak areas of SSP asphalt mastic at 1455 cm^−1^, 1375 cm^−1^, 719 cm^−1^, and 872 cm^−1^ are smaller than those of LP asphalt mastic. This observation indicates that SSP has adsorbed a greater amount of free aliphatic compounds, resulting in an increased relative content of heavy components in SSP asphalt mastic, which enhances its high-temperature performance. These findings align with the results of previous experiments. In summary, after mixing with asphalt, both LP and SSP fillers adsorb more polar molecules from the asphalt without disrupting the fillers’ crystalline structure or chemical bonds and without generating new functional groups. The interaction between the fillers and asphalt is limited to physical and chemical adsorption, with no evidence of a chemical reaction [35]. Although no chemical reactions occurred between LP or SSP and the asphalt, both fillers absorbed the polar components of the asphalt, indicating that the asphalt wrapped the crystalline structures of the two fillers, leading to a reduction in the FTIR absorption peaks. Furthermore, as shown in Figure 13, the peak intensity of SSP is lower than that of LP. This can be attributed to the fact that SSP has a specific surface area 7.06% higher than that of LP, resulting in a stronger adsorption capacity compared to LP.

As an advanced spectroscopic technique, two-dimensional infrared correlation spectroscopy (2D-COS) surpasses conventional one-dimensional FTIR in exploring structural and interactive changes within complex polymer systems under external perturbations at the molecular level. By extending the original FTIR into a second dimension, 2D-COS enhances the spectral resolution through correlations between distinct wavenumbers, thereby providing comprehensive insights into dynamic molecular interactions. Specifically, 2D-COS accurately resolves intermolecular interactions within polymer chains, such as van der Waals forces and hydrogen bonding, by identifying synchronized or asynchronous spectral responses under controlled perturbations.

**Figure 14:**
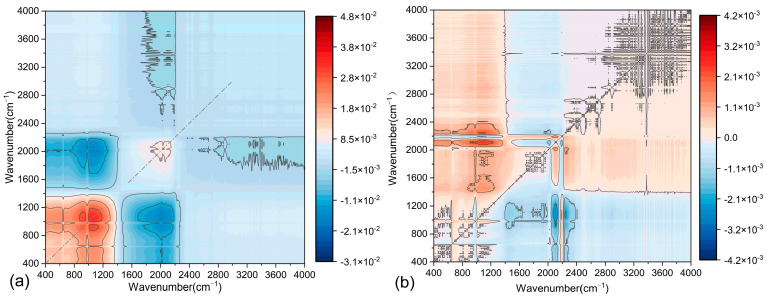
Two-dimensional infrared correlation spectroscopy results of LMA: (**a**) two-dimensional simultaneous correlation infrared spectra; (**b**) two-dimensional asynchronous correlation infrared spectra.

**Figure 15:**
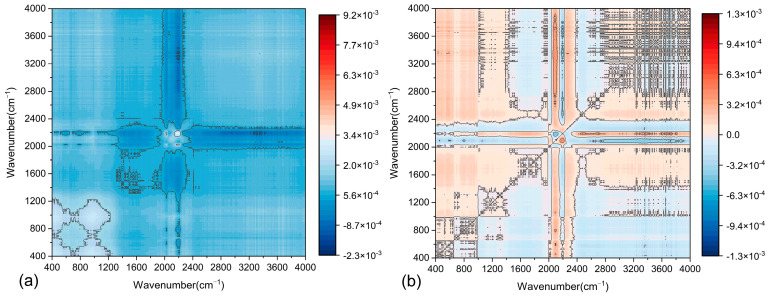
Two-dimensional infrared correlation spectroscopy results of SMA: (**a**) two-dimensional simultaneous correlation infrared spectra; (**b**) two-dimensional asynchronous correlation infrared spectra.

The application of 2D-COS to asphalt mastic enables the precise determination of functional group content variations and interaction heterogeneities under diverse conditions, providing critical insights into the physicochemical compatibility between asphalt and fillers. This approach effectively resolves dynamic molecular-level responses to external stimuli, thereby clarifying the evolution of interfacial interactions driven by hydrogen bonding or van der Waals forces. By using 2D-COS, the differences in functional group content and their interactions under various F/A ratios can be compared. This aids in understanding the physicochemical properties of the two asphalt mastics. In 2D-COS, the intensity of each pixel represents the interaction strength between different wavenumbers. In the synchronous spectrum, pixel intensities reflect the positive and negative correlations of vibrations associated with different chemical bonds. The asynchronous spectrum, on the other hand, reveals sequential relationships by analyzing dynamic changes occurring at different time points. As shown in Figure 14 and Figure 15, the synchronous 2D-COS spectra of the two asphalt mastics display auto-correlation peaks and cross-peaks along the diagonal and off-diagonal lines, respectively. These peaks indicate the positive and negative correlations in the spectral intensity variations in filler-related variables. In contrast, the asynchronous 2D-COS spectra exhibit cross-peaks along both the diagonal and off-diagonal lines. The appearance of these cross-peaks may be associated with correlations in functional group variations, which are attributed to intermolecular or intramolecular interactions.

From Figure 14a, it is evident that in the synchronous 2D-COS spectrum of LP-modified asphalt mastics, strong positive correlation peaks appear in the 800–1200 cm^−1^ range, with the peak intensity at 1093 cm^−1^ being the most prominent. This indicates that the functional groups in this region respond strongly to changes in the F/A ratio. The analysis suggests that significant interactions occur between LP and asphalt, particularly in the Si-O and Al-O bond regions. In contrast, distinct negative correlation peaks are observed at 2000 cm^−1^ and within the 2800–4000 cm^−1^ range, indicating that the functional groups in these regions are less affected by variations in LP content. Furthermore, the peak at 2920 cm^−1^ shows minimal variation, which is attributed to the antisymmetric stretching vibration absorption band of -CH_2_-. This suggests that LP primarily influences unsaturated hydrocarbons. Figure 13b shows the asynchronous 2D-COS spectrum of LMA. The results reveal sequential changes within the 800–1200 cm^−1^ region, suggesting that these changes are primarily driven by the initial distribution and diffusion of the filler. Additionally, several cross-peaks are observed, with the following four peak pairs showing the highest intensities: (1096, 1485 cm^−1^), (1120, 2000 cm^−1^), (1120, 2115 cm^−1^), and (1100, 2300 cm^−1^).

Figure 15 presents the 2D-COS spectrum of SMA. Compared to LMA, the positions of the auto-correlation peaks have shifted. In the synchronous 2D-COS spectrum, all auto-correlation peaks of SMA exhibit negative correlations, with the lowest intensities observed in the 400–1400 cm^−1^ range. Higher signal intensities are observed in the 1200–1600 cm^−1^ and 2800–3600 cm^−1^ ranges. These stronger signals are likely attributable to the effects of oxides (e.g., Fe-O, Ca-O) in the steel slag powder on the asphalt matrix. Distinct asynchronous signals are observed in the 2800–3600 cm^−1^ range, which are associated with the dynamic responses of C-H bond vibrations within the asphalt matrix. This observation suggests that the steel slag powder may induce a minor molecular rearrangement process within the asphalt matrix.

Figure 14 and Figure 15 indicate that the structure of LMA is predominantly enhanced through physical adsorption, while the SSP-modified asphalt mastic may involve trace chemical reactions (primarily physical adsorption) that lead to molecular rearrangement within the matrix. This process requires a longer duration to reach a stable state. Consequently, the 2DIR spectra of the two asphalt mastics exhibit distinct morphological characteristics. The synchronous spectrum of LMA demonstrates stronger physical interactions, with minimal dynamic changes observed in the asynchronous spectrum. In contrast, the synchronous spectrum of SMA reveals more pronounced chemical adsorption, while its asynchronous spectrum displays significant dynamic changes. This suggests a stronger molecular reorganization capability within the SMA.

**Figure 16:**
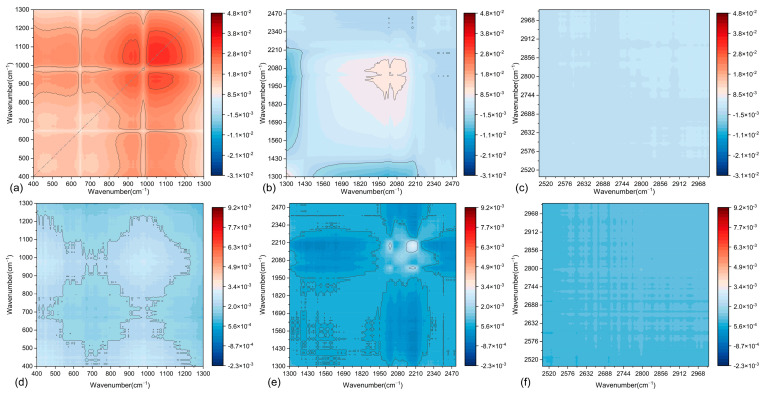
Two-dimensional simultaneous correlation infrared spectra at different wavenumbers: (**a**–**c**) LP asphalt mastic: 400~1300 cm^−1^, 1200~2400 cm^−1^, 2500~3000 cm^−1^; (**d**–**f**) SSP asphalt mastic: 400~1300 cm^−1^, 1200~2400 cm^−1^, 2500~3000 cm^−1^.

**Figure 17:**
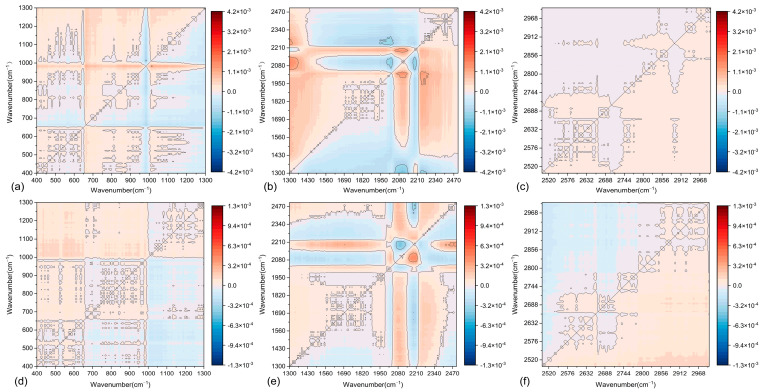
Two-dimensional asynchronous correlation infrared spectra at different wavenumbers: (**a**–**c**) LMA: 400~1300 cm^−1^, 1200~2400 cm^−1^, 2500~3000 cm^−1^; (**d**–**f**) SMA: 400~1300 cm^−1^, 1200~2400 cm^−1^, 2500~3000 cm^−1^.

In the original spectrum, numerous overlapping peaks are observed in the fingerprint region. Significant differences in the auto-correlation peaks of the spectra for the two asphalt mastics are identified within the 400–1300 cm^−1^, 1200–2400 cm^−1^, and 2500–3000 cm^−1^ intervals. To improve the resolution and obtain more detailed spectral information, characteristic extraction was performed for these three feature bands. This approach produced clearer synchronous and asynchronous 2D-COS spectra, as illustrated in Figure 16 and Figure 17.

**Figure 18:**
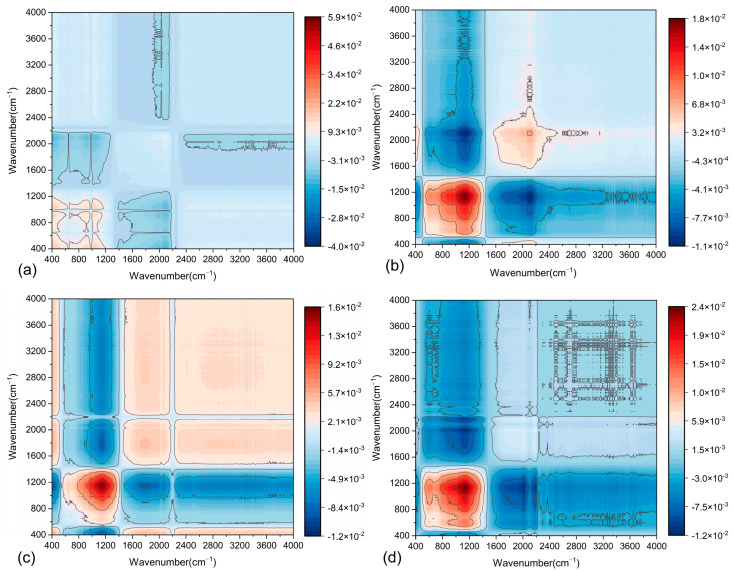
Two-dimensional infrared spectroscopy results of modified asphalt mastics: (**a**) F/A = 0.6; (**b**) F/A = 0.8; (**c**) F/A = 1.0; (**d**) F/A = 1.2.

To compare the effects of LP and SSP on the functional groups of asphalt mastics at the same F/A ratios, the synchronous 2D-COS spectra of the two fillers under four F/A ratios (0.6, 0.8, 1.0, and 1.2) were analyzed, as shown in Figure 18. The results reveal that as the F/A ratio increases, both the positions and intensities of the auto-peaks undergo significant changes. Specifically, the number of positive auto-peaks initially increases and then decreases, while the intensity of positive auto-peaks within the 400–1300 cm^−1^ range gradually strengthens. This indicates that the influence of LP and SSP on functional groups becomes more pronounced as the F/A ratio increases.

From Figure 18a–d, changes in the color intensity illustrate significant variations in specific chemical bonds or molecular groups within the samples. These variations are particularly evident in the 800–1200 cm^−1^ range (associated with mineral components) and the 2800–3600 cm^−1^ range (corresponding to the stretching vibrations of C-H bonds in the asphalt matrix). Additionally, the gradual enhancement in the intensity of positive auto-correlation peaks suggests that the addition of LP or SSP promotes the strengthening of specific intermolecular interactions. This may be related to particle-filling effects and their reinforcement of the asphalt matrix structure.

In the 800–1200 cm^−1^ range, the increase in peak intensity suggests that the Si-O and Al-O bonds in the mineral components of LP or SSP enhance the interactions between the particles and the asphalt matrix, leading to a significant filling effect. As the F/A ratio increases (see Figure 18d), the intensity of red peaks in this region becomes more pronounced, reflecting a higher proportion of particles relative to the matrix. In the 2800–3600 cm^−1^ range—which corresponds to the stretching vibrations of C-H bonds—the band may be influenced by interactions between the asphalt components and the surface substances of LP or SSP. Although the wavenumber changes remain relatively stable, the intensity increases at higher F/A ratios (F/A = 1.0 and F/A = 1.2). This suggests that the physical adsorption of the fillers may influence the saturated hydrocarbons and aromatic hydrocarbons within the asphalt matrix.

At low F/A ratios (0.6 and 0.8), the spectral signals are relatively dispersed, indicating weak interactions between LP or SSP and the matrix in the modified asphalt mastic. As the F/A ratio increased, the spectral signals became more concentrated with significantly enhanced intensity, indicating that the incorporation of filler markedly altered the intermolecular interaction patterns within the mastic, potentially inducing the formation of a more stable structure.

In both asphalt mastics, the positive auto-correlation peaks in the synchronous spectra exhibited enhanced intensity with increasing F/A ratio, indicating a denser microstructure and strengthened interactions between the filler and asphalt, which concomitantly promoted the development of a three-dimensional molecular network. Concurrently, the asynchronous spectra demonstrated a reduced variation in peak distribution, suggesting a progressive stabilization of structural homogeneity within the asphalt mastic at higher F/A ratios.

To further evaluate the potential of SSP as a novel filler, this study systematically summarizes the comprehensive performance of various new fillers based on recent research findings from other scholars, as shown in Table 8. By comparing the advantages and disadvantages of SSP, LP, fly ash (FA), and direct coal liquefaction residue (DCLR) in terms of high-temperature performance, adhesion capability, temperature sensitivity, environmental impact, and economic cost of the asphalt mastic, it was found that each type of filler possesses unique performance characteristics. For instance, LP exhibits the lowest cost, while FA and DCLR significantly enhance the high-temperature performance of asphalt mastic. However, when considering all evaluation metrics comprehensively, SSP not only effectively improves the performance of asphalt mastic but also demonstrates lower economic costs and excellent environmental sustainability, thereby enhancing the performance of asphalt mastic while reducing its environmental impact.

In asphalt mastic, although SSP demonstrates superior performance and better process compatibility compared to LP or other fillers, its regional supply limitations may hinder large-scale applications. To address this issue, future research plans will focus on exploring alternative supply strategies or developing hybrid filler systems to mitigate supply constraints and further optimize its application potential.

## 4. Conclusions

This study investigated the effect of SSP on the properties of asphalt mastic. Based on the test results, the following conclusions were drawn:

(1) Microstructural characterization revealed that SSP exhibits a superior specific surface area and distinctive internal porous architecture compared to LP. These morphological advantages significantly enhance the interfacial adsorption between SSP particles and the asphalt matrix;
(2) The rheological characterization revealed that the SMA mixture exhibited enhanced high-temperature rutting resistance and superior permanent deformation resistance. Notably, the incorporation of SSP as a filler effectively reduced the temperature susceptibility of asphalt mastics, demonstrating a reduction of 9.37–18.06% compared to LP while delivering superior filler reinforcement effects across varying stress levels (16.86–32.81% enhancement at 0.1 kPa and 7.91–24.52% improvement at 3.2 kPa for the SSP-modified system relative to LP);
(3) FTIR tests revealed that SSP, due to its larger specific surface area, can adsorb more polar molecules of asphalt, resulting in a reduction in absorption peaks and an improvement in the high-temperature performance of asphalt mastic. Further analysis using 2D-COS confirmed that the primary mechanism of interaction between the SSP, LP, and asphalt components is physical adsorption. These interfacial interactions gradually strengthen with an increase in the F/A ratio.

In conclusion, the strategic substitution of LP with SSP as a functional filler optimizes the high-temperature rheological performance of asphalt mastics. Implementing SSP-enhanced formulations in pavement materials for high-temperature regions offers a sustainable solution for steel slag valorization. This approach concurrently addresses environmental concerns through industrial by-product utilization and extends pavement service life by improving durability under thermal stress conditions.

## Figures and Tables

**Table 1:** Physical properties of 90# asphalt.

Technical Index	Request	Measured	Test Method
Penetration (25 °C)/mm	80–100	87	T 0604
Softening point/°C	≥45	45.3	T 0606
Ductility (10 °C)	≥45	>100	T 0605
Density/(g·cm^−3^)	-	0.983	T 0603
Flash point/°C	≥230	291	T 0611

**Table 2:** Physical properties of fillers.

Type	Apparent Relative Density (g/cm^−3^)	Relative Density to Water (g/cm^−3^)	Hydrophilicity	Water Content
LP	2.702	2.721	0.66	0.4
SSP	3.69	3.78	0.95	0.38

**Table 3:** Key indexes of particle size of different fillers.

Type	Average Surface Area Particle Size/μm	Volume Average Particle Size/μm	D_10_/μm	D_50_/μm	D_90_/μm	Specific Surface Area/(m^2^·g^−1^)
SSP	9.126	43.55	3.714	42.47	99.68	0.2607
LP	8.524	71.17	3.180	32.86	181.7	0.2435

**Table 4:** The physical properties of filler samples.

Samples	S_BET_ (m^2^·g^−1^)	Pore Volume (cm^2^·g^−1^)	Average Pore Size (nm)	Micropore Area (m^2^·g^−1^)
LP	2.3805	0.000014	20.6736	0.1457
SSP	1.8173	0.00001	17.4469	0.0870

**Table 5:** Results of G′ fitting of 8 asphalt mastics.

Type	y = a + bx	a	b	|b|	R^2^
SMA-0.6	y = 11.65 − 6.58x	11.65	−6.58	6.58	0.9697
SMA-0.8	y = 13.58 − 6.28x	13.58	−6.28	6.28	0.9995
SMA-1.0	y = 13.37 − 6.14x	13.37	−6.14	6.14	0.9993
SMA-1.2	y = 13.62 − 5.67x	13.62	−5.67	5.67	0.9933
LMA-0.6	y = 13.24 − 7.26x	13.24	−7.26	7.26	0.9953
LMA-0.8	y = 12.82 − 7.25x	12.82	−7.25	7.25	0.9983
LMA-1.0	y = 13.37 − 7.14x	13.37	−7.14	7.14	0.9994
LMA-1.2	y = 13.68 − 6.92x	13.68	−6.92	6.92	0.9982

**Table 6:** Results of G″ fitting of 8 asphalt mastics.

Type	y = a + bx	a	b	|b|	R^2^
SMA-0.6	y = 15.16 − 8.73x	15.16	−8.73	8.73	0.9995
SMA-0.8	y = 15.47 − 8.58x	15.47	−8.58	8.58	0.9985
SMA-1.0	y = 14.97 − 8.15x	14.97	−8.15	8.15	0.9911
SMA-1.2	y = 15.15 − 7.86x	15.15	−7.86	7.86	0.9971
LMA-0.6	y = 15.34 − 8.99x	15.34	−8.99	8.99	0.9943
LMA-0.8	y = 14.7 − 8.52x	14.7	−8.52	8.52	0.9959
LMA-1.0	y = 15.39 − 8.22x	15.39	−8.22	8.22	0.9998
LMA-1.2	y = 14.39 − 8.18x	14.39	−8.18	8.18	0.9960

**Table 7:** Results of MSCR test evaluation indicators.

Type	*J_nr_* (kPa^−1^)		*R* (%)		*R_diff_* (%)	*J_nr diff_* (%)
	100 Pa	3200 Pa	100 Pa	3200 Pa		
90# matrix asphalt	2.70	3.80	16.24	2.23	86.29	40.5
LMA(F/A = 0.6)	1.89	2.03	3.63	1.08	70.25	9.62
LMA (F/A = 0.8)	1.64	1.79	3.45	1.15	66.64	9.15
LMA (F/A = 1.0)	1.28	1.39	4.21	1.26	70.09	8.21
LMA (F/A = 1.2)	1.17	1.32	5.71	1.42	78.87	6.53
SMA(F/A = 0.6)	1.73	1.89	3.4	1.05	69.13	8.79
SMA (F/A = 0.8)	1.35	1.47	3.59	1.13	68.59	8.82
SMA (F/A = 1.0)	0.906	0.99	5.1	1.6	68.58	9.18
SMA (F/A = 1.2)	0.668	0.712	5.78	1.66	71.25	12.88

**Table 8:** Comprehensive performance comparison table of different fillers.

Category	Filler Type				
	SSP (This Study) [17,23,30]	LP [17,23,30]	CR+SSP [8]	Direct Coal Liquefaction Residue (DCLR) [36]	Fly Ash (FA) [36,37]
High-temperature performance	High	Low	High	High	High
Adsorption capacity	High	Low	High	Moderate	High
Temperature sensitivity	Decreased	-	Decreased	Decreased	-
Environmental impact	Low carbon footprint	Moderate (quarrying impacts)	Moderate	Low	Low carbon footprint
Cost	Low	Very low	Moderate	Low	Low

## Data Availability

The original contributions presented in this study are included in the article. Further inquiries can be directed to the corresponding author.

## Abbreviations

SSP	Steel slag powder
LP	Limestone powder
F/A	Filler-to-asphalt
SMA	SSP-modified asphalt mastic
LMA	LP-modified asphalt mastic
FTIR	Fourier-transform infrared spectroscopy
2D-COS	Two-dimensional correlation analysis
